# Octa­kis[2,2′,5,5′-tetra­thia­fulvalenium(0.5+)] bis­[hexa­molybdate(2−)] acetonitrile solvate

**DOI:** 10.1107/S1600536809031092

**Published:** 2009-08-12

**Authors:** Ikram Zebiri, Berkahoum Anak, Yacine Djebli, Sihem Boufas, Leïla Bencharif

**Affiliations:** a Laboratoire de Chimie des Matériaux, Faculté des Sciences, Université Mentouri, 25000 Constantine, Algeria; b Université 20 Aout 1955, Skikda, Algeria

## Abstract

The asymmetric unit of the title compound, (C_6_H_4_S_4_)_8_[Mo_6_O_19_]_2_·CH_3_CN, contains two halves of two centrosymmetric [Mo_6_O_19_]^2−^ hexa­molybdate anions, which are each built up from six distorted MoO_6_ octa­hedra sharing common edges and one common vertex at the central O atom, six tetra­thia­fulvalene cations (three of which are located on mirror planes) to balance the charge and a half of an acetonitrile solvent mol­ecule, likewise located on a mirror plane. The two central hexa­molybdate O atoms occupy special positions 2*a* and 2*d*, respectively. The cations and anions are inter­linked through C—H⋯O contacts.

## Related literature

For the chemical and physical properties of polyoxo­molybdates, see: Shi *et al.* (2006 ▶); Wang *et al.* (2004 ▶); Hagrman *et al.* (1999 ▶). For the structure of ammonium tris­(tetra­ethyl­ammonium) hexa­cosa­oxidoocta­molybdate, see: Zebiri *et al.* (2008 ▶). The title compound is isostructural with its tungsten analogue, see: Triki *et al.* (1993 ▶). The structure of the anions is the same as that in bis­[2-(pyrimidin-2-yl­amino)pyrimidinium] hexa­molybdate, see: Yeh *et al.* (2008 ▶). For Mo—O distances, see: Boyle *et al.* (1998 ▶); Deng *et al.* (2006 ▶); Maeda *et al.* (2006 ▶).
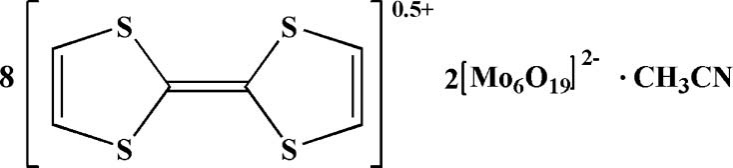


## Experimental

### 

#### Crystal data

(C_6_H_4_S_4_)_8_[Mo_6_O_19_]_2_·C_2_H_3_N
*M* *_r_* = 3434.99
Monoclinic, 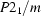

*a* = 14.3179 (8) Å
*b* = 20.2299 (10) Å
*c* = 16.7625 (10) Å
β = 101.266 (3)°
*V* = 4761.7 (5) Å^3^
*Z* = 2
Mo *K*α radiation
μ = 2.31 mm^−1^
*T* = 100 K
0.25 × 0.15 × 0.10 mm

#### Data collection

Nonius KappaCCD diffractometer
Absorption correction: multi-scan (*SADABS*; Sheldrick, 1996 ▶) *T*_min_ = 0.597, *T*_max_ = 0.802
45527 measured reflections
11170 independent reflections
8144 reflections with *I* > 2σ(*I*)
*R*_int_ = 0.072

#### Refinement

*R*[*F* ^2^ > 2σ(*F* ^2^)] = 0.036
*wR*(*F* ^2^) = 0.073
*S* = 1
11170 reflections
629 parameters
H-atom parameters constrained
Δρ_max_ = 0.88 e Å^−3^
Δρ_min_ = −0.70 e Å^−3^

### 

Data collection: *COLLECT* (Nonius, 2002 ▶); cell refinement: *DENZO* and *SCALEPACK* (Otwinowski & Minor, 1997 ▶); data reduction: *DENZO* and *SCALEPACK*; program(s) used to solve structure: *SIR92* (Altomare *et al.*, 1993 ▶); program(s) used to refine structure: *SHELXL97* (Sheldrick, 2008 ▶); molecular graphics: *ORTEP-3* (Farrugia, 1997 ▶) and *Mercury* (Macrae *et al.*, 2006 ▶); software used to prepare material for publication: *WinGX* (Farrugia, 1999 ▶) and *PARST* (Nardelli, 1995 ▶).

## Supplementary Material

Crystal structure: contains datablocks global, I. DOI: 10.1107/S1600536809031092/hg2545sup1.cif

Structure factors: contains datablocks I. DOI: 10.1107/S1600536809031092/hg2545Isup2.hkl

Additional supplementary materials:  crystallographic information; 3D view; checkCIF report

## Figures and Tables

**Table 1:** Hydrogen-bond geometry (Å, °)

*D*—H⋯*A*	*D*—H	H⋯*A*	*D*⋯*A*	*D*—H⋯*A*
C5—H5⋯O11^i^	0.93	2.28	3.192 (6)	167
C7—H7⋯O19^ii^	0.93	2.47	3.251 (7)	141
C7—H7⋯O19^iii^	0.93	2.47	3.251 (7)	141
C9—H9⋯O12	0.93	2.54	3.388 (5)	151
C13—H13⋯O17^iv^	0.93	2.41	3.239 (6)	149
C14—H14⋯O19^v^	0.93	2.59	3.175 (5)	121
C18—H18⋯O18^vi^	0.93	2.41	3.145 (5)	136
C19—H19⋯O10^vii^	0.93	2.41	3.298 (5)	160
C20—H20⋯O12	0.93	2.54	3.437 (5)	162
C24—H24⋯O8^viii^	0.93	2.25	3.075 (6)	148
C28—H28⋯O4^viii^	0.93	2.53	3.384 (6)	152
C29—H29⋯O5^viii^	0.93	2.52	3.256 (6)	137
C29—H29⋯O6^viii^	0.93	2.55	3.284 (6)	136

Symmetry codes: (i) 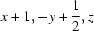; (ii) 
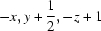
; (iii) 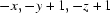; (iv) 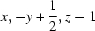; (v) 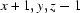; (vi) 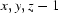; (vii) 
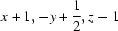
; (viii) 
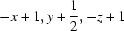
.

**Fig. 1.**
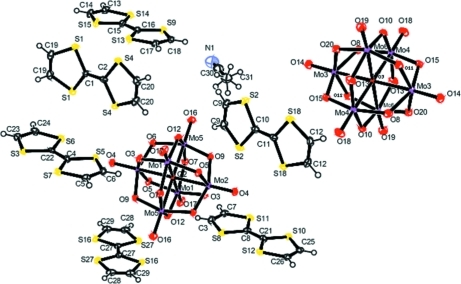
The independent components of (I), showing the atom-labelling scheme. Displacement ellipsoids are drawn at the 50% probability level.

**Fig. 2.**
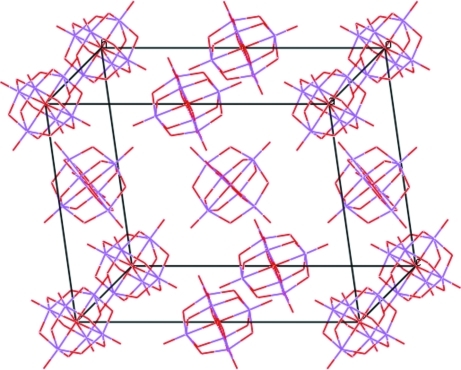
hexamolybdate anions positions on the unit cell.

## supplementary crystallographic information

### Comment

As part of an ongoing study of materials containing polyoxomolybdates, we have just recently determined the structure of ammonium tris(tetraethylammonium) hexacosaoxidooctamolybdate (Zebiri *et al.*, 2008). These materials show interesting chemical and physical properties (Shi *et al.*, 2006; Wang, *et al.*, 2004; Hagrman, *et al.*, 1999).

The asymmetric unit of the title compound consists of a half neutral acetonitrile; two hexamolybdate anions and six tetrathiafulvalene in witch two ones are (+1) charged and the rest ones with +0.5 charge (Fig. 1). The title compound is isostructural to (TTF)_4_W_6_O_19_.0.5(CH_3_CN) reported by Triki *et al.* (1993). The structure of the anions, as reported recently in Bis[2-(pyrimidin-2-ylamino)pyrimidinium] hexamolybdate (Yeh *et al.*, 2008), is constructed from an array of six edge-shared MoO_6_ octahedra with six O(*t*), ten O(µ2) and one O(µ6) atoms.

The Mo—O distances, ranging from 1.681 (3) to 2.3363 (4) Å, agree with those reported for other [Mo_6_O_19_]^2-^ anions in the literature (Deng *et al.*, 2006; Maeda *et al.*, 2006; Boyle *et al.*, 1998) and can be grouped into three sets bridging groups [Mo—O(terminal) 1.681 (3) -1.696 (3) Å, Mo—O(µ2): 1.850 (3) -2.027 (3) (1) Å and Mo—O(µ6): 2.3151 (4) (1)- 2.3363 (4) (1) Å.

Hexamolybdate anions spread along the *b* axis (Fig. 2) between which organic moieties intercalate. The cations and anions are interlinked through C—H···O contacts.

### Experimental

Single crystals of the title compound were prepared from a mixture of (NH_4_)_6_Mo_7_O_24_. 1.5 H_2_O (137 mg, 1 mmol), C_6_S_4_H_4_ (TTF) (612 mg, 3 mmol) and 3 ml H_2_O, heated in a Teflon-lined steel autoclave inside a programmable electric furnace at 160 ° C for 3 days. After cooling the autoclave to room temperature for 72 h, colorless crystals were obtained, filtered, washed with H_2_O, EtOH, Et_2_O and dried in air.

### Refinement

H atoms were positioned geometrically and treated as riding, with C—H = 0.93 Å with *U*_iso_(H) = 1.2Ueq(C). The methyl H atoms were constrained to an ideal geometry (C—H = 0.96 Å) with *U*_iso_(H) = 1.2*U*_eq_(C), but were allowed to rotate freely about the C—C bonds.

### Crystal data

**Table:**

(C_6_H_4_S_4_)_8_[Mo_6_O_19_]_2_·C_2_H_3_N	*F*(000) = 3324
*M**_r_* = 3434.99	*D*_x_ = 2.396 Mg m^−^^3^
Monoclinic, *P*2_1_/*m*	Mo *K*α radiation, λ = 0.71073 Å
Hall symbol: -P 2yb	Cell parameters from 2190 reflections
*a* = 14.3179 (8) Å	θ = 2.8–27.3°
*b* = 20.2299 (10) Å	µ = 2.31 mm^−^^1^
*c* = 16.7625 (10) Å	*T* = 100 K
β = 101.266 (3)°	Plates, colourless
*V* = 4761.7 (5) Å^3^	0.25 × 0.15 × 0.1 mm
*Z* = 2	

### Data collection

**Table:**

Nonius KappaCCD diffractometer	11170 independent reflections
Radiation source: fine-focus sealed X-ray tube	8144 reflections with *I* > 2σ(*I*)
graphite	*R*_int_ = 0.072
φ scans, and ω scans with κ offsets	θ_max_ = 27.5°, θ_min_ = 1.5°
Absorption correction: multi-scan (*SADABS*; Sheldrick, 1996)	*h* = −18→18
*T*_min_ = 0.597, *T*_max_ = 0.802	*k* = −26→26
45527 measured reflections	*l* = −21→21

### Refinement

**Table:**

Refinement on *F*^2^	Secondary atom site location: difference Fourier map
Least-squares matrix: full	Hydrogen site location: inferred from neighbouring sites
*R*[*F*^2^ > 2σ(*F*^2^)] = 0.036	H-atom parameters constrained
*wR*(*F*^2^) = 0.073	*w* = 1/[σ^2^(*F*_o_^2^) + (0.021*P*)^2^] where *P* = (*F*_o_^2^ + 2*F*_c_^2^)/3
*S* = 1	(Δ/σ)_max_ = 0.003
11170 reflections	Δρ_max_ = 0.88 e Å^−^^3^
629 parameters	Δρ_min_ = −0.70 e Å^−^^3^
0 restraints	Extinction correction: *SHELXL97* (Sheldrick, 2008), Fc^*^=kFc[1+0.001xFc^2^λ^3^/sin(2θ)]^-1/4^
Primary atom site location: structure-invariant direct methods	Extinction coefficient: 0.08 (2)

### Special details

**Table:**

Geometry. All e.s.d.'s (except the e.s.d. in the dihedral angle between two l.s. planes) are estimated using the full covariance matrix. The cell e.s.d.'s are taken into account individually in the estimation of e.s.d.'s in distances, angles and torsion angles; correlations between e.s.d.'s in cell parameters are only used when they are defined by crystal symmetry. An approximate (isotropic) treatment of cell e.s.d.'s is used for estimating e.s.d.'s involving l.s. planes.

### Fractional atomic coordinates and isotropic or equivalent isotropic displacement parameters (Å^2^)

**Table:**

	*x*	*y*	*z*	*U*_iso_*/*U*_eq_	Occ. (<1)
Mo1	0.56723 (3)	0.411518 (19)	0.58067 (2)	0.01535 (9)	
Mo2	0.37343 (3)	0.500847 (18)	0.56738 (2)	0.01262 (8)	
Mo5	0.41862 (3)	0.425991 (19)	0.40423 (2)	0.01562 (9)	
Mo3	0.09471 (3)	0.007942 (18)	0.90255 (2)	0.01440 (9)	
Mo4	0.12057 (3)	0.050345 (18)	1.09450 (2)	0.01550 (9)	
Mo6	−0.06607 (3)	0.102673 (18)	0.96110 (2)	0.01461 (9)	
S4	0.69926 (8)	0.32365 (5)	0.32774 (7)	0.0171 (2)	
S1	0.83864 (9)	0.32314 (6)	0.19478 (7)	0.0194 (3)	
S2	0.31973 (9)	0.32349 (5)	0.68472 (7)	0.0193 (3)	
S3	1.13888 (8)	0.41650 (5)	0.47871 (7)	0.0141 (2)	
S5	0.85700 (8)	0.41673 (5)	0.53863 (7)	0.0134 (2)	
S6	0.94719 (8)	0.41562 (5)	0.37833 (7)	0.0148 (2)	
S7	1.04691 (8)	0.41757 (5)	0.64209 (7)	0.0158 (2)	
S8	0.19893 (11)	0.75	0.39380 (10)	0.0162 (3)	
S9	0.38303 (8)	0.16665 (6)	0.03769 (7)	0.0186 (3)	
S10	−0.09582 (11)	0.75	0.43604 (10)	0.0167 (3)	
S11	0.01173 (12)	0.75	0.27956 (10)	0.0187 (4)	
S12	0.09410 (11)	0.75	0.54638 (9)	0.0157 (3)	
S13	0.57537 (8)	0.16549 (5)	0.13620 (7)	0.0169 (2)	
S14	0.47171 (8)	0.16799 (6)	−0.12637 (7)	0.0174 (2)	
S15	0.66417 (8)	0.16465 (6)	−0.02771 (7)	0.0204 (3)	
S18	0.21028 (8)	0.32348 (5)	0.83822 (7)	0.0177 (2)	
O1	0	0	1	0.0106 (9)	
O2	0.5	0.5	0.5	0.0119 (9)	
O5	0.4513 (2)	0.43340 (14)	0.62260 (17)	0.0153 (7)	
O4	0.2801 (2)	0.50318 (14)	0.61450 (18)	0.0159 (7)	
O3	0.6587 (2)	0.43007 (14)	0.51664 (18)	0.0169 (7)	
O6	0.4587 (2)	0.56620 (14)	0.62598 (17)	0.0150 (7)	
O12	0.4888 (2)	0.36829 (14)	0.49387 (18)	0.0161 (7)	
O7	0.6179 (2)	0.49293 (14)	0.64424 (17)	0.0166 (7)	
O8	−0.0228 (2)	−0.03840 (14)	0.84668 (18)	0.0195 (7)	
O9	0.3326 (2)	0.44003 (14)	0.47865 (18)	0.0159 (7)	
O10	0.0391 (2)	0.12578 (13)	1.03983 (18)	0.0166 (7)	
O11	0.0124 (2)	0.08657 (13)	0.88356 (18)	0.0178 (7)	
O13	0.1560 (2)	−0.03770 (14)	1.10491 (19)	0.0204 (7)	
O14	0.1549 (2)	0.01313 (14)	0.82549 (19)	0.0215 (7)	
O15	0.1685 (2)	0.05279 (13)	0.99087 (18)	0.0158 (7)	
O16	0.3610 (2)	0.36980 (15)	0.33835 (19)	0.0234 (8)	
O19	−0.1227 (2)	0.17476 (14)	0.93106 (19)	0.0221 (7)	
O18	0.2048 (2)	0.09129 (14)	1.1612 (2)	0.0245 (8)	
O17	0.6166 (2)	0.35055 (15)	0.64365 (19)	0.0245 (8)	
O20	0.1308 (2)	−0.07465 (14)	0.95163 (19)	0.0200 (7)	
C1	0.7991 (4)	0.25	0.2352 (4)	0.0127 (13)	
C4	0.9789 (3)	0.4167 (2)	0.5449 (3)	0.0145 (9)	
C3	0.1983 (5)	0.75	0.2897 (4)	0.0204 (15)	
H3	0.2548	0.75	0.2703	0.025*	
C2	0.7423 (4)	0.25	0.2902 (4)	0.0143 (13)	
C7	0.1143 (5)	0.75	0.2388 (4)	0.0224 (15)	
H7	0.1107	0.75	0.1828	0.027*	
C8	0.0738 (5)	0.75	0.3820 (4)	0.0170 (14)	
C6	0.8635 (3)	0.4170 (2)	0.6423 (3)	0.0166 (10)	
H6	0.8088	0.4169	0.6643	0.02*	
C5	0.9505 (3)	0.4174 (2)	0.6901 (3)	0.0178 (10)	
H5	0.9585	0.4176	0.7465	0.021*	
C11	0.2420 (4)	0.25	0.7930 (4)	0.0149 (13)	
C10	0.2854 (4)	0.25	0.7292 (4)	0.0160 (14)	
C15	0.5424 (3)	0.1666 (2)	−0.0301 (3)	0.0174 (10)	
C16	0.5044 (3)	0.1662 (2)	0.0406 (3)	0.0172 (10)	
C13	0.5655 (3)	0.1660 (2)	−0.1775 (3)	0.0216 (11)	
H13	0.5553	0.166	−0.234	0.026*	
C12	0.1572 (3)	0.2831 (2)	0.9098 (3)	0.0223 (11)	
H12	0.1297	0.3068	0.9468	0.027*	
C14	0.6539 (3)	0.1643 (2)	−0.1321 (3)	0.0210 (11)	
H14	0.7074	0.163	−0.1559	0.025*	
C9	0.3514 (3)	0.2832 (2)	0.6016 (3)	0.0231 (11)	
H9	0.3676	0.307	0.5588	0.028*	
C22	1.0177 (3)	0.41639 (19)	0.4749 (3)	0.0132 (9)	
C17	0.4818 (3)	0.1637 (2)	0.1873 (3)	0.0180 (10)	
H17	0.4923	0.1623	0.2438	0.022*	
C18	0.3931 (3)	0.1643 (2)	0.1421 (3)	0.0185 (10)	
H18	0.3396	0.1635	0.1659	0.022*	
C21	0.0295 (4)	0.75	0.4457 (4)	0.0165 (14)	
C20	0.6422 (3)	0.2833 (2)	0.3968 (3)	0.0211 (11)	
H20	0.6127	0.307	0.4325	0.025*	
C19	0.9255 (3)	0.2830 (2)	0.1533 (3)	0.0235 (11)	
H19	0.9704	0.3067	0.1319	0.028*	
C24	1.0417 (3)	0.4110 (2)	0.3287 (3)	0.0197 (10)	
H24	1.0322	0.4084	0.2723	0.024*	
C23	1.1297 (3)	0.4114 (2)	0.3748 (3)	0.0174 (10)	
H23	1.1836	0.4089	0.3516	0.021*	
C26	−0.0077 (4)	0.75	0.5908 (4)	0.0184 (14)	
H26	−0.0027	0.75	0.6469	0.022*	
C25	−0.0915 (5)	0.75	0.5408 (4)	0.0186 (14)	
H25	−0.1474	0.75	0.5613	0.022*	
S16	0.43075 (9)	0.99818 (6)	0.10594 (7)	0.0200 (3)	
S27	0.63331 (9)	0.99422 (6)	0.09037 (7)	0.0203 (3)	
C27	0.5135 (3)	0.9988 (2)	0.0403 (3)	0.0166 (10)	
C29	0.5178 (4)	0.9954 (2)	0.1949 (3)	0.0245 (11)	
H29	0.5008	0.9954	0.2457	0.029*	
C28	0.6083 (4)	0.9931 (2)	0.1884 (3)	0.0247 (11)	
H28	0.6568	0.991	0.2342	0.03*	
N1	0.1799 (6)	0.25	0.1110 (5)	0.061 (2)	
C30	0.1822 (6)	0.25	0.1791 (7)	0.049 (3)	
C31	0.1876 (6)	0.25	0.2689 (6)	0.050 (2)	
H31A	0.1353	0.2749	0.2815	0.076*	0.5
H31B	0.2466	0.2697	0.2953	0.076*	0.5
H31C	0.1845	0.2054	0.2876	0.076*	0.5

### Atomic displacement parameters (Å^2^)

**Table:**

	*U*^11^	*U*^22^	*U*^33^	*U*^12^	*U*^13^	*U*^23^
Mo1	0.0150 (2)	0.0195 (2)	0.0125 (2)	0.00474 (16)	0.00522 (16)	0.00528 (16)
Mo2	0.01036 (18)	0.0189 (2)	0.00921 (18)	0.00057 (16)	0.00335 (15)	0.00047 (15)
Mo5	0.0138 (2)	0.0217 (2)	0.01209 (19)	−0.00355 (16)	0.00420 (16)	−0.00492 (16)
Mo3	0.0168 (2)	0.0162 (2)	0.01122 (19)	−0.00087 (16)	0.00517 (16)	0.00087 (15)
Mo4	0.0164 (2)	0.0170 (2)	0.01202 (19)	−0.00345 (16)	0.00029 (16)	−0.00233 (15)
Mo6	0.0185 (2)	0.0131 (2)	0.01240 (19)	0.00125 (16)	0.00348 (16)	0.00127 (15)
S4	0.0199 (6)	0.0162 (6)	0.0154 (6)	0.0025 (5)	0.0038 (5)	−0.0010 (5)
S1	0.0213 (6)	0.0191 (6)	0.0189 (6)	−0.0011 (5)	0.0062 (5)	0.0029 (5)
S2	0.0241 (7)	0.0177 (6)	0.0166 (6)	−0.0050 (5)	0.0055 (5)	0.0010 (5)
S3	0.0110 (5)	0.0164 (6)	0.0148 (6)	−0.0019 (4)	0.0023 (5)	−0.0003 (4)
S5	0.0114 (5)	0.0163 (6)	0.0122 (5)	0.0001 (4)	0.0018 (4)	0.0009 (4)
S6	0.0148 (6)	0.0171 (6)	0.0117 (5)	−0.0021 (4)	0.0006 (5)	0.0014 (4)
S7	0.0152 (6)	0.0186 (6)	0.0121 (5)	−0.0005 (5)	−0.0008 (5)	0.0000 (4)
S8	0.0155 (8)	0.0175 (8)	0.0151 (8)	0	0.0017 (7)	0
S9	0.0149 (6)	0.0223 (6)	0.0184 (6)	−0.0005 (5)	0.0028 (5)	−0.0022 (5)
S10	0.0151 (8)	0.0176 (8)	0.0161 (8)	0	−0.0006 (7)	0
S11	0.0227 (9)	0.0184 (8)	0.0124 (8)	0	−0.0027 (7)	0
S12	0.0155 (8)	0.0173 (8)	0.0126 (8)	0	−0.0016 (7)	0
S13	0.0163 (6)	0.0215 (6)	0.0127 (6)	0.0001 (5)	0.0021 (5)	−0.0004 (5)
S14	0.0188 (6)	0.0213 (6)	0.0118 (6)	0.0011 (5)	0.0019 (5)	−0.0001 (5)
S15	0.0158 (6)	0.0278 (7)	0.0182 (6)	0.0030 (5)	0.0046 (5)	0.0017 (5)
S18	0.0210 (6)	0.0147 (6)	0.0177 (6)	−0.0002 (5)	0.0046 (5)	−0.0021 (5)
O1	0.013 (2)	0.012 (2)	0.007 (2)	−0.0012 (17)	0.0024 (17)	−0.0010 (16)
O2	0.010 (2)	0.016 (2)	0.011 (2)	0.0002 (17)	0.0043 (18)	0.0001 (17)
O5	0.0175 (17)	0.0193 (16)	0.0103 (15)	0.0010 (13)	0.0054 (13)	0.0021 (12)
O4	0.0135 (16)	0.0218 (17)	0.0133 (16)	−0.0001 (13)	0.0047 (13)	−0.0004 (13)
O3	0.0134 (16)	0.0221 (16)	0.0164 (17)	0.0041 (13)	0.0052 (14)	0.0028 (13)
O6	0.0153 (16)	0.0199 (16)	0.0109 (15)	−0.0017 (13)	0.0053 (13)	−0.0024 (13)
O12	0.0207 (17)	0.0149 (16)	0.0149 (16)	−0.0004 (13)	0.0091 (14)	−0.0004 (12)
O7	0.0131 (16)	0.0259 (17)	0.0102 (16)	0.0016 (13)	0.0012 (13)	0.0014 (13)
O8	0.0240 (18)	0.0230 (17)	0.0114 (16)	−0.0007 (14)	0.0032 (14)	−0.0036 (13)
O9	0.0137 (16)	0.0207 (17)	0.0149 (16)	−0.0036 (13)	0.0068 (13)	−0.0031 (13)
O10	0.0193 (17)	0.0122 (15)	0.0188 (17)	−0.0036 (13)	0.0049 (14)	−0.0019 (13)
O11	0.0200 (17)	0.0164 (16)	0.0168 (17)	−0.0021 (13)	0.0026 (14)	0.0020 (13)
O13	0.0192 (17)	0.0219 (17)	0.0173 (17)	−0.0010 (14)	−0.0034 (14)	0.0015 (14)
O14	0.0254 (19)	0.0192 (17)	0.0229 (19)	−0.0013 (14)	0.0120 (16)	0.0011 (14)
O15	0.0126 (16)	0.0171 (16)	0.0183 (17)	−0.0035 (13)	0.0040 (13)	−0.0032 (13)
O16	0.0220 (18)	0.0321 (19)	0.0167 (18)	−0.0051 (15)	0.0054 (15)	−0.0125 (14)
O19	0.0264 (19)	0.0181 (17)	0.0227 (18)	0.0025 (14)	0.0066 (16)	0.0043 (14)
O18	0.0244 (18)	0.0251 (18)	0.0215 (18)	−0.0084 (14)	−0.0017 (15)	−0.0043 (14)
O17	0.028 (2)	0.0275 (18)	0.0197 (19)	0.0115 (15)	0.0097 (16)	0.0075 (14)
O20	0.0219 (18)	0.0160 (16)	0.0245 (19)	−0.0015 (13)	0.0102 (15)	−0.0005 (14)
C1	0.015 (3)	0.013 (3)	0.011 (3)	0	0.006 (3)	0
C4	0.011 (2)	0.012 (2)	0.019 (2)	0.0005 (17)	−0.0005 (19)	0.0019 (18)
C3	0.022 (4)	0.028 (4)	0.012 (3)	0	0.006 (3)	0
C2	0.018 (3)	0.014 (3)	0.009 (3)	0	−0.002 (3)	0
C7	0.036 (4)	0.021 (4)	0.012 (3)	0	0.010 (3)	0
C8	0.026 (4)	0.019 (3)	0.005 (3)	0	−0.001 (3)	0
C6	0.016 (2)	0.017 (2)	0.018 (2)	0.0003 (18)	0.006 (2)	0.0009 (19)
C5	0.026 (3)	0.017 (2)	0.011 (2)	−0.002 (2)	0.005 (2)	−0.0006 (18)
C11	0.014 (3)	0.015 (3)	0.014 (3)	0	−0.003 (3)	0
C10	0.016 (3)	0.015 (3)	0.015 (3)	0	0.000 (3)	0
C15	0.016 (2)	0.021 (2)	0.014 (2)	0.0026 (19)	0.001 (2)	−0.0020 (19)
C16	0.019 (2)	0.018 (2)	0.015 (2)	−0.0004 (19)	0.004 (2)	−0.0005 (19)
C13	0.032 (3)	0.021 (3)	0.014 (2)	0.005 (2)	0.010 (2)	0.0023 (19)
C12	0.019 (3)	0.028 (3)	0.021 (3)	0.005 (2)	0.009 (2)	−0.001 (2)
C14	0.020 (3)	0.028 (3)	0.017 (3)	0.006 (2)	0.010 (2)	0.001 (2)
C9	0.024 (3)	0.031 (3)	0.016 (3)	−0.003 (2)	0.008 (2)	0.002 (2)
C22	0.014 (2)	0.007 (2)	0.016 (2)	−0.0029 (17)	−0.0017 (19)	−0.0001 (17)
C17	0.023 (3)	0.019 (2)	0.013 (2)	0.001 (2)	0.007 (2)	−0.0021 (19)
C18	0.021 (3)	0.016 (2)	0.022 (3)	−0.0033 (19)	0.011 (2)	−0.0040 (19)
C21	0.014 (3)	0.018 (3)	0.013 (3)	0	−0.006 (3)	0
C20	0.024 (3)	0.030 (3)	0.010 (2)	0.006 (2)	0.007 (2)	−0.0007 (19)
C19	0.023 (3)	0.034 (3)	0.017 (3)	−0.003 (2)	0.011 (2)	0.001 (2)
C24	0.025 (3)	0.022 (3)	0.011 (2)	−0.005 (2)	0.002 (2)	0.0009 (19)
C23	0.021 (2)	0.020 (2)	0.012 (2)	−0.0021 (19)	0.005 (2)	0.0018 (18)
C26	0.021 (4)	0.015 (3)	0.020 (4)	0	0.005 (3)	0
C25	0.027 (4)	0.013 (3)	0.017 (3)	0	0.006 (3)	0
S16	0.0263 (7)	0.0219 (6)	0.0124 (6)	0.0039 (5)	0.0053 (5)	−0.0005 (5)
S27	0.0219 (6)	0.0245 (6)	0.0127 (6)	0.0035 (5)	−0.0012 (5)	−0.0004 (5)
C27	0.021 (2)	0.015 (2)	0.013 (2)	0.0028 (19)	0.003 (2)	−0.0002 (18)
C29	0.039 (3)	0.022 (3)	0.010 (2)	0.002 (2)	−0.001 (2)	−0.0004 (19)
C28	0.035 (3)	0.022 (3)	0.012 (2)	0.005 (2)	−0.007 (2)	−0.0015 (19)
N1	0.071 (6)	0.060 (5)	0.050 (5)	0	0.002 (5)	0
C30	0.038 (5)	0.026 (5)	0.084 (8)	0	0.014 (6)	0
C31	0.051 (6)	0.033 (5)	0.071 (7)	0	0.021 (5)	0

### Geometric parameters (Å, °)

**Table:**

Mo1—O17	1.686 (3)	S15—C14	1.727 (5)
Mo1—O12	1.872 (3)	S15—C15	1.736 (5)
Mo1—O3	1.886 (3)	S18—C12	1.743 (5)
Mo1—O5	1.974 (3)	S18—C11	1.768 (4)
Mo1—O7	2.018 (3)	O1—Mo6^ii^	2.3222 (4)
Mo1—O2	2.3345 (4)	O1—Mo3^ii^	2.3234 (4)
Mo2—O4	1.680 (3)	O1—Mo4^ii^	2.3363 (4)
Mo2—O5	1.887 (3)	O2—Mo2^i^	2.3151 (4)
Mo2—O9	1.931 (3)	O2—Mo5^i^	2.3335 (4)
Mo2—O6	1.932 (3)	O2—Mo1^i^	2.3345 (4)
Mo2—O3^i^	1.974 (3)	O3—Mo2^i^	1.974 (3)
Mo2—O2	2.3151 (4)	O6—Mo5^i^	1.928 (3)
Mo5—O16	1.684 (3)	O7—Mo5^i^	1.860 (3)
Mo5—O7^i^	1.860 (3)	O8—Mo4^ii^	1.879 (3)
Mo5—O6^i^	1.928 (3)	O13—Mo6^ii^	2.013 (3)
Mo5—O9	1.937 (3)	O20—Mo6^ii^	1.962 (3)
Mo5—O12	2.010 (3)	C1—C2	1.343 (8)
Mo5—O2	2.3335 (4)	C1—S1^iii^	1.766 (3)
Mo3—O14	1.690 (3)	C4—C22	1.393 (6)
Mo3—O15	1.876 (3)	C3—C7	1.331 (9)
Mo3—O20	1.889 (3)	C3—H3	0.93
Mo3—O11	1.968 (3)	C2—S4^iii^	1.774 (4)
Mo3—O8	1.993 (3)	C7—H7	0.93
Mo3—O1	2.3234 (4)	C8—C21	1.345 (9)
Mo4—O18	1.692 (3)	C6—C5	1.343 (6)
Mo4—O13	1.851 (3)	C6—H6	0.93
Mo4—O8^ii^	1.879 (3)	C5—H5	0.93
Mo4—O15	1.989 (3)	C11—C10	1.339 (9)
Mo4—O10	2.028 (3)	C11—S18^iii^	1.768 (4)
Mo4—O1	2.3363 (4)	C10—S2^iii^	1.774 (4)
Mo6—O19	1.696 (3)	C15—C16	1.398 (6)
Mo6—O10	1.858 (3)	C13—C14	1.343 (6)
Mo6—O11	1.905 (3)	C13—H13	0.93
Mo6—O20^ii^	1.962 (3)	C12—C12^iii^	1.340 (9)
Mo6—O13^ii^	2.013 (3)	C12—H12	0.93
Mo6—O1	2.3222 (4)	C14—H14	0.93
S4—C20	1.746 (5)	C9—C9^iii^	1.343 (9)
S4—C2	1.774 (4)	C9—H9	0.93
S1—C19	1.739 (5)	C17—C18	1.347 (6)
S1—C1	1.766 (3)	C17—H17	0.93
S2—C9	1.749 (5)	C18—H18	0.93
S2—C10	1.774 (4)	C20—C20^iii^	1.347 (9)
S3—C23	1.723 (4)	C20—H20	0.93
S3—C22	1.724 (4)	C19—C19^iii^	1.335 (9)
S5—C6	1.721 (5)	C19—H19	0.93
S5—C4	1.727 (4)	C24—C23	1.344 (6)
S6—C24	1.724 (5)	C24—H24	0.93
S6—C22	1.732 (4)	C23—H23	0.93
S7—C4	1.728 (4)	C26—C25	1.323 (9)
S7—C5	1.728 (5)	C26—H26	0.93
S8—C3	1.743 (6)	C25—H25	0.93
S8—C8	1.764 (7)	S16—C29	1.748 (5)
S9—C18	1.728 (5)	S16—C27	1.767 (5)
S9—C16	1.728 (5)	S27—C28	1.749 (5)
S10—C25	1.746 (6)	S27—C27	1.759 (5)
S10—C21	1.769 (6)	C27—C27^iv^	1.330 (8)
S11—C7	1.737 (7)	C29—C28	1.322 (7)
S11—C8	1.772 (6)	C29—H29	0.93
S12—C21	1.759 (6)	C28—H28	0.93
S12—C26	1.761 (7)	N1—C30	1.136 (12)
S13—C16	1.722 (5)	C30—C31	1.492 (13)
S13—C17	1.725 (5)	C31—H31A	0.96
S14—C15	1.729 (4)	C31—H31B	0.96
S14—C13	1.729 (5)	C31—H31C	0.96
			
O17—Mo1—O12	105.04 (14)	Mo2—O2—Mo5^i^	89.589 (13)
O17—Mo1—O3	104.43 (14)	Mo2^i^—O2—Mo5^i^	90.411 (14)
O12—Mo1—O3	91.61 (13)	Mo2—O2—Mo5	90.411 (14)
O17—Mo1—O5	102.70 (14)	Mo2^i^—O2—Mo5	89.589 (13)
O12—Mo1—O5	87.90 (12)	Mo5^i^—O2—Mo5	180.000 (15)
O3—Mo1—O5	152.02 (12)	Mo2—O2—Mo1	89.800 (13)
O17—Mo1—O7	101.69 (14)	Mo2^i^—O2—Mo1	90.200 (13)
O12—Mo1—O7	152.99 (12)	Mo5^i^—O2—Mo1	89.969 (14)
O3—Mo1—O7	85.42 (12)	Mo5—O2—Mo1	90.031 (14)
O5—Mo1—O7	82.51 (12)	Mo2—O2—Mo1^i^	90.200 (13)
O17—Mo1—O2	176.68 (11)	Mo2^i^—O2—Mo1^i^	89.800 (13)
O12—Mo1—O2	77.94 (9)	Mo5^i^—O2—Mo1^i^	90.031 (14)
O3—Mo1—O2	76.77 (9)	Mo5—O2—Mo1^i^	89.969 (14)
O5—Mo1—O2	75.78 (8)	Mo1—O2—Mo1^i^	180
O7—Mo1—O2	75.24 (8)	Mo2—O5—Mo1	116.42 (14)
O4—Mo2—O5	103.76 (13)	Mo1—O3—Mo2^i^	117.16 (14)
O4—Mo2—O9	103.37 (13)	Mo5^i^—O6—Mo2	116.11 (14)
O5—Mo2—O9	88.74 (12)	Mo1—O12—Mo5	116.50 (14)
O4—Mo2—O6	102.89 (13)	Mo5^i^—O7—Mo1	116.59 (14)
O5—Mo2—O6	89.50 (12)	Mo4^ii^—O8—Mo3	117.46 (15)
O9—Mo2—O6	153.33 (12)	Mo2—O9—Mo5	117.05 (14)
O4—Mo2—O3^i^	102.71 (13)	Mo6—O10—Mo4	116.53 (14)
O5—Mo2—O3^i^	153.52 (12)	Mo6—O11—Mo3	116.24 (15)
O9—Mo2—O3^i^	84.73 (12)	Mo4—O13—Mo6^ii^	116.26 (15)
O6—Mo2—O3^i^	85.07 (12)	Mo3—O15—Mo4	116.10 (14)
O4—Mo2—O2	178.36 (10)	Mo3—O20—Mo6^ii^	116.81 (15)
O5—Mo2—O2	77.87 (9)	C2—C1—S1	123.08 (17)
O9—Mo2—O2	76.54 (9)	C2—C1—S1^iii^	123.08 (17)
O6—Mo2—O2	77.07 (9)	S1—C1—S1^iii^	113.8 (3)
O3^i^—Mo2—O2	75.66 (8)	C22—C4—S5	120.9 (3)
O16—Mo5—O7^i^	104.41 (14)	C22—C4—S7	123.4 (3)
O16—Mo5—O6^i^	103.77 (14)	S5—C4—S7	115.7 (3)
O7^i^—Mo5—O6^i^	90.34 (12)	C7—C3—S8	118.0 (5)
O16—Mo5—O9	103.27 (14)	C7—C3—H3	121
O7^i^—Mo5—O9	89.55 (12)	S8—C3—H3	121
O6^i^—Mo5—O9	152.09 (12)	C1—C2—S4^iii^	122.83 (18)
O16—Mo5—O12	101.98 (14)	C1—C2—S4	122.83 (18)
O7^i^—Mo5—O12	153.60 (12)	S4^iii^—C2—S4	114.3 (4)
O6^i^—Mo5—O12	84.01 (12)	C3—C7—S11	118.3 (5)
O9—Mo5—O12	83.78 (12)	C3—C7—H7	120.8
O16—Mo5—O2	177.36 (11)	S11—C7—H7	120.8
O7^i^—Mo5—O2	78.16 (9)	C21—C8—S8	122.5 (5)
O6^i^—Mo5—O2	76.70 (8)	C21—C8—S11	123.0 (5)
O9—Mo5—O2	75.98 (8)	S8—C8—S11	114.5 (4)
O12—Mo5—O2	75.45 (8)	C5—C6—S5	117.6 (4)
O14—Mo3—O15	106.28 (14)	C5—C6—H6	121.2
O14—Mo3—O20	104.51 (14)	S5—C6—H6	121.2
O15—Mo3—O20	91.14 (13)	C6—C5—S7	117.0 (4)
O14—Mo3—O11	101.82 (14)	C6—C5—H5	121.5
O15—Mo3—O11	87.94 (12)	S7—C5—H5	121.5
O20—Mo3—O11	152.81 (13)	C10—C11—S18^iii^	122.77 (18)
O14—Mo3—O8	100.35 (14)	C10—C11—S18	122.77 (18)
O15—Mo3—O8	153.12 (13)	S18^iii^—C11—S18	114.5 (4)
O20—Mo3—O8	85.58 (13)	C11—C10—S2	123.05 (18)
O11—Mo3—O8	83.12 (12)	C11—C10—S2^iii^	123.05 (18)
O14—Mo3—O1	175.05 (11)	S2—C10—S2^iii^	113.8 (4)
O15—Mo3—O1	78.15 (9)	C16—C15—S14	122.5 (3)
O20—Mo3—O1	77.32 (9)	C16—C15—S15	122.4 (3)
O11—Mo3—O1	75.90 (9)	S14—C15—S15	115.1 (3)
O8—Mo3—O1	75.10 (9)	C15—C16—S13	122.1 (4)
O18—Mo4—O13	105.18 (14)	C15—C16—S9	122.2 (4)
O18—Mo4—O8^ii^	102.97 (15)	S13—C16—S9	115.7 (3)
O13—Mo4—O8^ii^	92.59 (13)	C14—C13—S14	117.2 (4)
O18—Mo4—O15	103.98 (14)	C14—C13—H13	121.4
O13—Mo4—O15	88.36 (13)	S14—C13—H13	121.4
O8^ii^—Mo4—O15	151.77 (12)	C12^iii^—C12—S18	117.92 (15)
O18—Mo4—O10	101.72 (13)	C12^iii^—C12—H12	121
O13—Mo4—O10	152.84 (12)	S18—C12—H12	121
O8^ii^—Mo4—O10	84.97 (12)	C13—C14—S15	117.2 (4)
O15—Mo4—O10	81.52 (12)	C13—C14—H14	121.4
O18—Mo4—O1	176.53 (11)	S15—C14—H14	121.4
O13—Mo4—O1	78.29 (9)	C9^iii^—C9—S2	117.77 (15)
O8^ii^—Mo4—O1	76.83 (9)	C9^iii^—C9—H9	121.1
O15—Mo4—O1	75.75 (8)	S2—C9—H9	121.1
O10—Mo4—O1	74.81 (8)	C4—C22—S3	122.2 (3)
O19—Mo6—O10	105.57 (14)	C4—C22—S6	122.1 (3)
O19—Mo6—O11	104.81 (14)	S3—C22—S6	115.7 (3)
O10—Mo6—O11	91.35 (13)	C18—C17—S13	117.3 (4)
O19—Mo6—O20^ii^	101.65 (14)	C18—C17—H17	121.3
O10—Mo6—O20^ii^	88.78 (13)	S13—C17—H17	121.3
O11—Mo6—O20^ii^	152.48 (12)	C17—C18—S9	117.0 (4)
O19—Mo6—O13^ii^	100.43 (14)	C17—C18—H18	121.5
O10—Mo6—O13^ii^	153.81 (12)	S9—C18—H18	121.5
O11—Mo6—O13^ii^	84.97 (13)	C8—C21—S12	121.4 (5)
O20^ii^—Mo6—O13^ii^	82.91 (13)	C8—C21—S10	123.7 (5)
O19—Mo6—O1	175.58 (11)	S12—C21—S10	115.0 (4)
O10—Mo6—O1	78.25 (9)	C20^iii^—C20—S4	117.88 (15)
O11—Mo6—O1	77.08 (8)	C20^iii^—C20—H20	121.1
O20^ii^—Mo6—O1	76.01 (9)	S4—C20—H20	121.1
O13^ii^—Mo6—O1	75.65 (8)	C19^iii^—C19—S1	117.84 (15)
C20—S4—C2	94.7 (2)	C19^iii^—C19—H19	121.1
C19—S1—C1	94.0 (2)	S1—C19—H19	121.1
C9—S2—C10	94.6 (2)	C23—C24—S6	117.3 (4)
C23—S3—C22	94.9 (2)	C23—C24—H24	121.4
C6—S5—C4	94.8 (2)	S6—C24—H24	121.4
C24—S6—C22	94.7 (2)	C24—C23—S3	117.4 (4)
C4—S7—C5	94.9 (2)	C24—C23—H23	121.3
C3—S8—C8	94.6 (3)	S3—C23—H23	121.3
C18—S9—C16	95.0 (2)	C25—C26—S12	117.1 (5)
C25—S10—C21	94.1 (3)	C25—C26—H26	121.4
C7—S11—C8	94.5 (3)	S12—C26—H26	121.4
C21—S12—C26	94.7 (3)	C26—C25—S10	119.1 (5)
C16—S13—C17	95.0 (2)	C26—C25—H25	120.4
C15—S14—C13	95.3 (2)	S10—C25—H25	120.4
C14—S15—C15	95.2 (2)	C29—S16—C27	94.5 (2)
C12—S18—C11	94.8 (2)	C28—S27—C27	95.1 (2)
Mo6—O1—Mo6^ii^	180.000 (19)	C27^iv^—C27—S27	123.3 (5)
Mo6—O1—Mo3^ii^	89.861 (13)	C27^iv^—C27—S16	122.4 (5)
Mo6^ii^—O1—Mo3^ii^	90.139 (13)	S27—C27—S16	114.3 (2)
Mo6—O1—Mo3	90.139 (13)	C28—C29—S16	118.5 (4)
Mo6^ii^—O1—Mo3	89.861 (13)	C28—C29—H29	120.8
Mo3^ii^—O1—Mo3	180.0000 (10)	S16—C29—H29	120.8
Mo6—O1—Mo4	90.402 (14)	C29—C28—S27	117.5 (4)
Mo6^ii^—O1—Mo4	89.598 (13)	C29—C28—H28	121.3
Mo3^ii^—O1—Mo4	90.504 (14)	S27—C28—H28	121.3
Mo3—O1—Mo4	89.496 (14)	N1—C30—C31	178.7 (11)
Mo6—O1—Mo4^ii^	89.598 (13)	C30—C31—H31A	109.5
Mo6^ii^—O1—Mo4^ii^	90.402 (14)	C30—C31—H31B	109.5
Mo3^ii^—O1—Mo4^ii^	89.496 (14)	H31A—C31—H31B	109.5
Mo3—O1—Mo4^ii^	90.504 (14)	C30—C31—H31C	109.5
Mo4—O1—Mo4^ii^	180.0000 (10)	H31A—C31—H31C	109.5
Mo2—O2—Mo2^i^	180	H31B—C31—H31C	109.5

Symmetry codes: (i) −*x*+1, −*y*+1, −*z*+1; (ii) −*x*, −*y*, −*z*+2; (iii) *x*, −*y*+1/2, *z*; (iv) −*x*+1, −*y*+2, −*z*.

### Hydrogen-bond geometry (Å, °)

**Table:**

*D*—H···*A*	*D*—H	H···*A*	*D*···*A*	*D*—H···*A*
C5—H5···O11^v^	0.93	2.28	3.192 (6)	167
C7—H7···O19^vi^	0.93	2.47	3.251 (7)	141
C7—H7···O19^vii^	0.93	2.47	3.251 (7)	141
C9—H9···O12	0.93	2.54	3.388 (5)	151
C13—H13···O17^viii^	0.93	2.41	3.239 (6)	149
C14—H14···O19^ix^	0.93	2.59	3.175 (5)	121
C18—H18···O18^x^	0.93	2.41	3.145 (5)	136
C19—H19···O10^xi^	0.93	2.41	3.298 (5)	160
C20—H20···O12	0.93	2.54	3.437 (5)	162
C24—H24···O8^xii^	0.93	2.25	3.075 (6)	148
C28—H28···O4^xii^	0.93	2.53	3.384 (6)	152
C29—H29···O5^xii^	0.93	2.52	3.256 (6)	137
C29—H29···O6^xii^	0.93	2.55	3.284 (6)	136

Symmetry codes: (v) *x*+1, −*y*+1/2, *z*; (vi) −*x*, *y*+1/2, −*z*+1; (vii) −*x*, −*y*+1, −*z*+1;  (viii) *x*, −*y*+1/2, *z*−1; (ix) *x*+1, *y*, *z*−1; (x) *x*, *y*, *z*−1; (xi) *x*+1, −*y*+1/2, *z*−1; (xii) −*x*+1, *y*+1/2, −*z*+1.
